# Detection of zeb1 Gene in Granulosa Cells in Women Undergoing IVF Treatment

**DOI:** 10.3390/jcm12175652

**Published:** 2023-08-30

**Authors:** Ioannis Chrysanthopoulos, Despoina Mavrogianni, Eirini Drakaki, Anastasios Potiris, Athanasios Zikopoulos, Athanasios Zachariou, Ekaterini Domali, Peter Drakakis, Sofoklis Stavros

**Affiliations:** 1 First Department of Obstetrics and Gynecology, Alexandra Hospital, Medical School, National and Kapodistrian University of Athens, 115 28 Athens, Greece; johnchrysan@med.uoa.gr (I.C.); dmavrogianni@med.uoa.gr (D.M.); eirinidrak@med.uoa.gr (E.D.); kdomali@med.uoa.gr (E.D.); pdrakakis@med.uoa.gr (P.D.); 2 Third Department of Obstetrics and Gynecology, University General Hospital “ATTIKON”, Medical School, National and Kapodistrian University of Athens, 12462 Athens, Greece; sfstavrou@med.uoa.gr; 3 Department of Obstetrics and Gynecology, Royal Cornwall Hospital, Treliske, Truro TR1 3LQ, UK; thanzik92@gmail.com; 4 Laboratory of Spermatology, Department of Urology, School of Health Sciences, University of Ioannina, 451 10 Ioannina, Greece; zahariou@otenet.gr

**Keywords:** infertility, in vitro fertilization (IVF), ZEB1 gene, granulosa cells, body mass index (BMI), age, oocyte number, oocyte maturation

## Abstract

Background: ZEB1 plays a role in epithelial-to-mesenchymal transition and acts as a repressor of E-cadherin, TGF-β, and Wnt/β-catenin. Since ZEB1 protein is expressed in estrogen-responsive tissues, and expression of the gene in the normal ovary and endometrium is positively correlated with high estrogen levels, we performed a direct analysis of granulosa cell samples to determine whether there are any significant changes in zeb1 expression during folliculogenesis. Methods: ZEB1 expression levels were measured in the granulosa cells of 56 infertile women undergoing IVF treatment. RNA extraction from granulosa cells was performed along with reverse transcription quantitative polymerase chain reaction (RT-qPCR) with SYBR Green I to determine zeb1 gene expression levels. Statistical analysis was performed by using *t*-test, while possible correlations of the expression of ZEB1 protein with body mass index (BMI), age, number of oocytes, and oocyte maturation were investigated. Results: Zeb1 gene expression levels correlate significantly with body mass index (BMI) and age, but not with oocyte number and oocyte maturation stage. Obese women demonstrate a higher expression level of zeb1 gene compared to normal and overweight women. Moreover, zeb1 gene is overexpressed in women aged 35–40 years old and is under-expressed in women >40 years old. Conclusions: ZEB1 expression should be further investigated as it may unveil new potential findings of the zeb1 gene’s role in female fertility and its use as a biomarker in fertility workups.

## 1. Introduction

The current clinical definition of infertility is the inability to conceive after 12 months of systematic and unprotected coitus. Infertility prevalence is approximately 13% among women and 10% among men. It is estimated through demographic data that 9% of couples worldwide lack the ability to conceive naturally, and approximately 180 million couples are characterized by infertility problems [1,2]. There are several causal and contributory factors that include hormonal inadequacies or imbalances, pathophysiological deviation in the reproductive anatomy and function, genetics, and lifestyle factors among others, while a significant percentage of infertility is attributed to unknown or idiopathic factors [3].

ZEB1 is a protein encoded by the zeb1 gene which is located on chromosome 10p11.2. In humans, ZEB1 protein is expressed in estrogen-responsive tissues such as the breast, bone, uterus, endometrium, ovary, and cardiovascular system. The overexpression of the gene in normal ovary and endometrium is correlated with high estrogen levels [4].

The zinc-finger E homeobox (ZEB) protein family of transcription factors consists of two members: ZEB1 and ZEB2 [5]. The ZEB family, as a transcription factor, regulates its transcription by binding to the E-promoter DNA sequence (5′-CANNTG-3′), In humans, ZEB1 contains more than a thousand amino acids and consists of two zinc-finger clusters at the N- and C-terminal ends of the protein. The middle region consists of a homeodomain, a Smad interaction domain, and a C-terminal binding protein (CtBP) interaction domain. CtBP cannot directly bind to DNA and participates in the regulation of ZEB1 function by interacting with other regulatory factors [6,7].

Epithelial-to-mesenchymal transition (EMT) defines a process during which cells lose their epithelial characteristics and acquire typical properties of mesenchymal cells. This transition requires complex changes in cell shape that happen concomitantly to gene expression reprogramming [8]. While EMT is an essential process during embryogenesis for delamination of neural crest from the neural tube, it is a major pathological event in cancer progression, causing invasion and metastasis, which are the primary causes of morbidity and mortality in human cancer [4].

The main hallmark of EMT is the downregulation of the adherens junction protein E-cadherin due to transcriptional repression [8]. Downregulation of E-cadherin, and the resulting release of cell–cell adhesion in tumors, is thought to contribute to a motile phenotype that facilitates metastasis. Conversely, mesenchymal genes including vimentin and smooth muscle actin, as well as various matrix and matrix-degrading enzymes, are induced during EMT [9]. In addition, it has been reported that the ZEB1 protein appears to inhibit the expression of cell polarity factors, suppresses basement membrane synthesis, and activates the expression of basement membrane metalloproteases (matrix metalloproteases (MMPs), such as MMP-1, MMP-9, and MMP-14. It also promotes basement membrane remodeling and invasion into adjacent tissues. The functions of ZEB1 protein are not limited in regulating the EMT, they are also considered a central switch that controls many cellular functions such as differentiation, proliferation, response to DNA damage, and cell survival with a strong impact on tumor growth in the early stages of tumorigenesis [10].

EMT-inducing pathways, such as transforming growth factor-β (TGFβ) and Wnt signaling trigger epithelial dedifferentiation by impairing the expression/ function of the epithelial adhesion protein E-cadherin [6,9,11,12]. Repression of E-cadherin is mediated by distinct transcription factors, including Snail1 (Snail), Snail2 (Slug), and SIP1(ZEB2). ZEB1 is also tightly controlled by micro-RNAs such as miR-200 (along with miR-200 family members miR-200a, miR-200c, and miR-141), which are involved in a negative feedback loop to control EMT during normal development and tumorigenesis [13].

Both transcription and post-transcriptional mechanisms are used to regulate Zeb1. Snail1 and TGF-β lower the levels of miR-200, which stabilizes ZEB1 RNA and Zeb1 protein. A second effect of EMT is the stimulation of ZEB1 gene transcription, which is dependent on the actions of Twist and Ets1. Snail1 boosts the function of these two factors in a unique way. More specifically, it increases Twist protein levels and facilitates Ets1 translocation to the nucleus. As a result, there is an increase in the binding of these two proteins to the ZEB1 promoter. Numerous effects of this multiple upregulation of zeb1 gene expression are possible. Consequently, it is proposed that Zeb1 upregulation might be cell-dependent. Thus, whereas the effect might be mainly transcriptional in some cells, the downregulation of miRNA might be predominant in other cells [8]. EMT is one of the main mechanisms involved in both cancer and endometriosis [9]. 

Endometriosis is one of the main causes of female infertility and increases the risk of ovarian cancer in nearly 10% of women in reproductive age [14]. One of the main mechanisms involved in both cancer and endometriosis is the epithelial-mesenchymal transition (EMT) [9]. The main fact of the epithelial-to-mesenchymal transition (EMT) is the down regulation of E-cadherin, a protein that plays a major role in cell-to-cell adhesion. ZEB1 protein is the main regulator of EMT, through direct or indirect inhibitory action on various downstream targets such as E-cadherin [7,10]. ZEB1 protein is expressed at high levels in the epithelial cells of endometriotic tissue, while it is not expressed in normal endometrium, which makes the epithelial expression of ZEB1 a hallmark of endometriosis [15,16].

Considering the relationship between the transcription factor ZEB1 in EMT and its expression levels in women with endometriosis, we decided to study its expression in granulosa cells derived from infertile women undergoing IVF treatment. Moreover, regarding the relationship between age and infertility, it has been shown that fertility decreases with increasing age in both men and women. However, the risk of infertility has a strong correlation with maternal age. Reproductive aging is a normal process of declining fertility as a woman goes through the stages of puberty, reproductive age, transition to menopause, and menopause [17].

The aim of the present study is to focus on the interaction between zeb1 gene expression and oocyte characteristics. More specifically, we try to determine whether there is any significance in zeb1 expression during folliculogenesis and, overall, the female fertility dynamics, since ZEB1 protein is expressed in estrogen-responsive tissues such as the breast, uterus, endometrium, and ovary, while expression of the gene in the normal ovary and endometrium is positively correlated with high estrogen levels.

## 2. Materials and Methods

### 2.1. Study Design, Population, and Participant Characteristics

The study was conducted in accordance with the Declaration of Helsinki and approved by the Institutional Review Board of the Medical School of the National and Kapodistrian University of Athens with protocol identifier 56353/26.11.2020. The study was conducted in the Assisted Reproduction Unit of the First Department of Obstetrics and Gynecology through a 7-month period. The data were retrospectively collected from 56 women diagnosed with infertility, aged 20–47 years old, who underwent IVF treatment. Informed consent was obtained from all patients involved in the study during their second visit in the Unit. For each patient, data were collected regarding body mass index (BMI), age, oocyte number, and oocyte maturation. The women were separated in three groups according to their BMI: normal weight, BMI = 18.5–24.99 kg/m^2^ (*n =* 38); overweight, BMI = 25.00–29.99 kg/m^2^ (*n =* 11); obese, BMI ≥30.00 kg/m^2^ (*n =* 7). Underweight women (BMI < 18.5 kg/m^2^) were not detected in our study. The division of these BMI groups was carried out in accordance with World Health Organization (WHO) criteria. 

For the comparison of zeb1 gene expression according to age, the women were separated into three groups: Group A, 20–34 years old (*n =* 23); Group B, 35–40 years old (*n =* 17); Group C, over 40 years old (*n =* 16). Female participants had an average age of 34.85 years (min = 20 years; max = 47 years) and median age of 37 years. We also performed a secondary analysis to investigate the effect of zeb1 expressed according to ovarian stimulation response; thus, we divided the cohort into normal responders and poor responders. According to the number of oocytes resulting from the ovarian response, women were separated in two groups: normal responders (*n =* 46) including women with more than 4 oocytes, and poor responders (*n =* 10) including women with less than 3 oocytes. Regarding oocyte maturation, the 56 women were separated in two groups: Group 1 with oocyte maturation 30–75% (*n = 19*), and Group 2 with oocyte maturation over 75% (*n =* 37). Cases with oocyte maturation less than 30% were not detected in this study. All groups (age, responders, and oocyte maturation) were divided according to Bologna and Poseidon criteria [18,19].

Exclusion criteria included female participants with any endometrial or endocrinological pathology and/or any medical history of endometriosis, hydrosalpinx, or autoimmune disorders.

### 2.2. Oocyte Retrieval, Denudation, and Maturity Evaluation 

Pituitary suppression was achieved by daily injection of 0.25 mg gonadotrophin-releasing hormone (GnRH) antagonist (Orgalutran; Organon, Oss, Netherlands, Cetrotide; Merck, Merk KGaA, Darmstadt, Germany). Gonadotropin stimulation consisted of recombinant FSH (Gonal F, Merk KGaA, Darmstadt, Germany; or Puregon (MSD, Kenilworth, NJ, USA), alone or in combination with urinary gonadotropins (hMG) (Menopur, Ferring, Saint-Prex Switzerland). Ovarian response was monitored by transvaginal ultrasound measurements of follicular growth and of serum estradiol and progesterone levels every 1–3 days. FSH and hMG dosages were adjusted accordingly. The trigger for final oocyte maturation with 250 μg of human chorionic gonadotrophin (Ovitrelle, Merck Serono Europe Limited, London, UK) was administrated when the two leading follicles reached a diameter of ≥17 mm. The dosage of the HCG trigger was determined on a sliding scale based on the estradiol levels of the patient. Transvaginal oocyte retrieval was performed under conscious sedation 35–37 h after HCG administration. Following oocyte aspiration procedure, oocytes were incubated in pre-equilibrated culture medium dishes (Universal IVF Medium, Origio a/s, Malov, Denmark). For the determination of ZEB1 expression, cumulus cells were collected on the day of oocyte retrieval. More specifically, the cells were segregated from cumulus oocyte complexes (COCs) by applying the process of stripping using 40 IU/mL hyaluronidase (Cumulase) [20]. To enhance the enzymatic removal of the cumulus and corona cells, the oocytes were aspirated in and out of a calibrated pipette stripper with an approximate inner dimeter of 200 μm. 

The oocyte maturation/nuclear maturity is morphologically computable, and it is validated by the extrusion of the first polar body [21]. After the removal of the cumulus/corona cells around the oocytes, these cells were kept in a deep freezer at −80 °C.

### 2.3. RNA Isolation and Reverse Transcription 

Total RNA was extracted from granulosa cells using Monarch Total RNA Miniprep Kit (New England Biolabs Inc., Ipswich, MA, USA). Single-stranded complementary DNA (cDNA) was synthetized from 100 ng of total RNA as a template using LunaScript RT SuperMix Kit (New England Biolabs Inc.) and according to the manufacturer’s protocol.

### 2.4. Quantitative Real-Time PCR (qRT-PCR) Analysis

The real-time quantitative PCR reaction was performed using a SYBR Luna Universal qPCR Master Mix kit (New England Biolabs Inc.) and the Light Cycler 480II (Roche Molecular Biochemicals, Mannheim Germany), according to the manufacturers protocol. The sequences of the qRT-PCR primer were as follows: zeb1 forward (5′-CAGCCCTGCAGTCCAAGAAC-3′), zeb1 reverse (5′-TTGTCTTTCATCCTGATTTCCATTT-3′), G6PD forward (5′-TGGACCTGACCTACGGCAACAGATA-3′), and G6PD reverse (5′-GCCCTCATACTGGAAACCC-3′). The qRT-PCR reaction was 30 s at 95 °C, 1 cycle, 5 s at 95 °C, and 30 s at 60 °C, 40 cycles. The 2^−ΔΔCT^ method was used to calculate the relative transcript abundance. All qRT-PCR reactions were repeated twice.

### 2.5. Statistical Analysis

Data are presented as the mean ± standard error (SE). Comparisons of dCt between groups were performed using Student’s F-test to ascertain if the variances were equal or unequal and then using a two-sample *t*-test assuming equal variances or unequal variances. Results were considered statistically significant when reaching *p* < 0.05 (5%) or tended to be statistically significant when reaching *p* < 0.1 (10%).

## 3. Results

### 3.1. Correlation of Zeb1 Expression Levels with BMI

Obese women with BMI ≥ 30.00 kg/m^2^ seemed to have a higher dCt compared to the other two groups. Obese women demonstrated a higher expression level of zeb1 gene compared women with normal weight, BMI = 18.5–24.99 kg/m^2^ (*p =* 0.036) and overweight women with statistically significant differences (*p =* 0.055). Women with normal weight and overweight women had no statistically significant difference in zeb1 expression levels (*p =* 0.385) (Table 1).

### 3.2. Correlation of Zeb1 Expression Levels with Age

Women aged 35–40 years old had higher zeb1 expression levels in the granulosa cell samples following oocyte retrieval, compared to other age groups. Moreover, women over 40 years old had reduced expression levels of zeb1 gene compared to the other age groups (Table 2). Comparing two age groups, 35–40 years and >40 years, zeb1 gene was overexpressed in women aged 35–40 years old and under-expressed in women >40 years old, and this result was statistically significant (*p =* 0.014 < 0.05) (Table 2).

The difference in expression levels of zeb1 gene between women aged 20–34 years old and >40 years old tended to be statistically significant (*p =* 0.097 < 0.1). However, between women aged 20–34 years old and 35–40 years old, the difference in zeb1 gene expression levels was not statistically significant (*p =* 0.14 > 0.05) (Table 2).

### 3.3. Zeb1 Expression Levels in Granulosa Cells

The correlation of Zeb1 expression with the ovarian response was also studied. Women were divided into two groups: normal responders and poor responders. After the comparison of mean dCt of these two groups, we found no statistically significant difference in zeb1 expression levels between normal responders and poor responders (*p =* 0.34) (Table 3). We also investigated if there was a correlation between zeb1 gene expression levels in granulosa cells and oocyte maturation. As shown in Table 4, there was no significant difference in mean dCt when comparing Group 1 with Group 2 (*p =* 0.263); thus, zeb1 gene expression levels seemed not to affect oocyte maturation rate.

## 4. Discussion

Infertility is the state of a diminished capacity to conceive and bear offspring. It is estimated to affect 8–12% of couples of reproductive age [22]. Endometriosis is one of the main causes of female infertility and increases the risk of ovarian cancer in nearly 10% of women of reproductive age [14]. ZEB1 protein is expressed at high levels in the epithelial cells of endometriotic tissue, while it is not expressed in the normal endometrium, which makes the epithelial expression of ZEB1 a hallmark of endometriosis [15,16]. 

One of the main mechanisms involved in both cancer and endometriosis is the epithelial-to-mesenchymal transition (EMT) [9]. The main aspect of epithelial-to-mesenchymal transition (EMT) is the downregulation of E-cadherin, a protein that plays a major role in cell-to-cell adhesion. ZEB1 protein is the main regulator of the progress of EMT, from its initiation and tumorigenesis up to metastasis through direct or indirect inhibitory action on various downstream targets such as E-cadherin [4,9,11]. 

On the basis of the above conclusions, regarding the role of the transcription factor ZEB1 in EMT and the relation of its expression levels in women with endometriosis, which makes them infertile, we decided to study the expression levels of this specific protein in granulosa cells derived from infertile women undergoing IVF. 

Regarding the relationship between age and infertility, it has been shown that fertility decreases with increasing age in both men and women. However, the risk of infertility has a strong correlation with maternal age. Accordingly, reproductive aging is a normal process of declining fertility [17]. Interestingly, we found that, in women aged 35–40 years, zeb1 gene had increased expression, while, in women over 40 years old, its expression levels decreased. 

Furthermore, it has been proven that BMI and obesity have a strong association with infertility. Studies have also shown that, in obese women, the fertility rate decreases with a simultaneous increase in BMI, while obese women have a three times greater risk of infertility compared to non-obese women [23,24]. We demonstrated that zeb1 gene had increased expression levels in granulosa cells of obese women compared to overweight and normal-weight women. 

Obesity also appears to affect oocyte quality by altering oocyte maturation and developmental capacity [23]. Our study also demonstrated that the expression of zeb1 gene could not directly affect the oocyte number and, therefore, the response of each woman to ovarian stimulation, nor did the maturation rate of the retrieved oocytes seem to correlate with zeb1 gene expression.

Zinc finger E-box binding homeobox 1 (ZEB1) promotes epithelial-to-mesenchymal transition (EMT) in carcinogenesis, but its role in embryo implantation has not yet been identified. The initial stage allowing the blastocyst to adhere to the endometrium during implantation is the migration of endometrial epithelial cells (EECs). During the mid-secretory phase of the menstrual cycle, ZEB1 was significantly expressed in the human endometrium at both mRNA and protein levels. ZEB1 knockdown prevented in vitro embryo implantation while altering EMT markers. ZEB1 may modify endometrial receptivity by promoting EMT, which may be essential for the embryo implantation process [25].

After parturition, menstruation, and, in certain cases, injury, the human uterine endometrium experiences considerable remodeling and regeneration on a regular basis. Successful human reproduction depends on the adult endometrium’s capacity for cyclic regeneration, differentiation, and decidualization. The endometrium must undergo processes known as mesenchymal-to-epithelial transition (MET) and epithelial-to-mesenchymal transition (EMT) in order for its cells to switch between mesenchymal and epithelial phenotypes. MET/EMT processes have been extensively studied in the context of cancer and embryonic development, but growing evidence shows how crucial they are for giving the endometrium the phenotypic and functional flexibility required for successful decidualization, regeneration/re-epithelialization, and embryo implantation [26,27].

Endometrial stromal fibroblasts develop epithelioid phenotypes in response to estrogen, progesterone, and numerous other stimuli. The maternal luminal epithelium and the decidualized stromal cells acquire mesenchymal characteristics of increased migration/motility during embryo implantation in response to both maternal and embryonic-derived signals undergoing EMT in order to accommodate the invading trophoblast [27]. Regulation of the EMT/MET processes required in the maternal endometrium during implantation depends on communication between the maternal and embryonic compartments. Molecular signals from the mother’s endometrium that have been linked to unsuccessful embryo implantation have also been demonstrated to control the EMT procedure. Most notably, microRNAs (miRNAs) have been found to play a role. Many aspects of embryo implantation have also been linked to miRNAs [28].

Additionally, compared to the peri-implantation interval, miR-30a-3p is considerably downregulated throughout the implantation period. Its expression depends on the status and presence of blastocysts in addition to endometrial decidualization. Furthermore, Snail2 expression appears to be reverse-regulated by miR-30a-3p. While downregulation accelerates embryo implantation by inducing EMT, upregulation of miR-30a-3p significantly decreases implantation sites both in vivo and in vitro. Thus, dysregulation of miR-30a-3p alters the ability of cells to migrate and invade by affecting EMT-related indicators, such as Zeb1 protein [29]. Therefore, miR-429, another miRNA, has an impact on embryo implantation. A gain of function for MiR-429 significantly decreases the number of implantation sites while having little effect on fertilization. MiR-429 suppresses migratory and invasive ability, as well as mesenchymal markers, which are indicators of miR-429’s ability to decrease the EMT in vivo and in vitro. Therefore, miR-429 downregulation may help in embryo implantation by promoting EMT [30].

In conclusion, our study demonstrated that the expression levels of the transcription factor ZEB1 in granulosa cells are influenced by two factors. Firstly, BMI, mainly in obese women, plays an important role, as the zeb1 gene demonstrates a statistically significant higher expression level compared to women with normal weight and overweight women. In addition, the age of women affects zeb1 gene expression levels, with the main changes being found in women over 35 years old. It is proposed to further explore ZEB1 protein expression levels, as its expression in estrogen-responsive tissues and its correlation with BMI and age may unveil new potential findings of the zeb1 gene’s role in female fertility and its use as a surrogate biomarker in fertility workups.

## Figures and Tables

**Table 1:** Mean dCt of each BMI group and its correlation with zeb1 gene expression levels.

BMI Group	Mean dCt
Normal	1.147631579
Overweight	1.405454545
Obese	3.18
**Compared Groups**	***p*-Value (5%)**
Normal vs. obese	0.0364868
Overweight vs. obese women	0.0558143
Normal vs. overweight	0.3874018

**Table 2:** Mean dCt of each age group and its correlation with zeb1 gene expression levels.

Age Group	Mean dCt
Group A (20–34 years)	1.44913043
Group B (35–40 years)	2.41411765
Group C (>40 years)	0.41625
**Compared Groups**	***p*-Value (5%)**
Group A—Group B	0.14036174
Group A—Group C	0.09724657
Group B—Group C	0.01430555

**Table 3:** Correlation of Zeb1 expression with oocyte number.

Groups Based on Oocyte Number	Mean dCt
Normal responders	1.515869565
Poor responders	1.15
**Compared Groups**	***p*-Value (5%)**
Normal responders vs. poor responders	0.3467274

**Table 4:** Correlation of Zeb1 expression with oocyte maturation.

Groups Based on Oocyte Maturation (%)	Mean dCt
Group 1 (30–75%)	1.13947368
Group 2 (>75%)	1.61297297
**Compared Groups**	***p*-Value (5%)**
Group 1 vs. Group 2	0.26379416

## Data Availability

Not applicable.
